# Maternal Antibiotic Exposure and the Risk of Developing Antenatal or Postpartum Depressive Symptoms: The Maternal Experience Study Protocol

**DOI:** 10.3390/mps6050098

**Published:** 2023-10-11

**Authors:** Mahsa Pouranayatihosseinabad, Maggie Taylor, Jason Hawrelak, Gregory M. Peterson, Felicity Veal, Tristan Ling, Mackenzie Williams, Megan Whatley, Kyan Ahdieh, Corinne Mirkazemi

**Affiliations:** 1 School of Pharmacy and Pharmacology, College of Health and Medicine, University of Tasmania, Hobart, TAS 7005, Australia; 2 Department of Obstetrics and Gynaecology, Royal Hobart Hospital, Hobart, TAS 7000, Australia; 3 Launceston Medical Centre, Health Hub, Launceston, TAS 7250, Australia

**Keywords:** antenatal, antenatal depression, antibiotic, association, depression, mental health, perinatal, postnatal, postpartum depression, pregnancy

## Abstract

Limited epidemiological evidence suggests a link between antibiotic use and developing depression. This study seeks to investigate this association in depth, using a cohort of pregnant individuals. The primary aim is to explore any association between the use of antibiotics during pregnancy and the development of antenatal depressive symptoms up to the third trimester, as well as the use of antibiotics during pregnancy and within 12 months postpartum and the development of postpartum depressive symptoms. A national prospective, observational, longitudinal cohort study has been designed to examine these relationships. A sample size of 1500 pregnant individuals has been sought for this study, assuming 10 potential predictor variables (including antibiotic use) in the final multiple logistic regression model and allowing for a 30% drop-out rate. The development of depressive symptoms is considered either a diagnosis by a medical doctor and/or a scoring 13 or higher on the Edinburgh Postnatal Depression Scale. Data will be collected during the third trimester and at 6 weeks, 6 months, and 12 months postpartum. These surveys include variables previously identified as associated with antenatal and postpartum depression (e.g., level of social support, experience of intimate partner abuse, and obstetric complications), as well as antibiotic and probiotic use. This study will provide an update on the prevalence of the symptoms of depression during pregnancy and postpartum and its associated risk factors. It will also, for the first time, comprehensively explore the potential association between antibiotic use during pregnancy and up to 12 months postpartum and the development of depressive symptoms.

## 1. Introduction

Depression is a leading cause of global disability, with an estimated 4.4% of the world’s population affected [1]. In Australia, it is estimated that 10.4% of individuals had depression or feelings of depression in 2017–2018, with more women experiencing symptoms than men (11.6% vs. 9.1%) [2]. Several mechanisms are postulated to be involved in its pathophysiology. Of these, the gastrointestinal (GI) microbiota dysbiosis theory is increasingly garnering attention [3,4]. Prior studies indicate that the risk of developing depression may increase by up to 20% following antibiotic exposure [5,6]. This risk may increase further with the number of antibiotic courses and agents used and slowly reduce over the following ten years [5]. Although relatively consistent in their findings, there have been few studies exploring this association, each with inherent limitations related to their methodologies [7]. 

To explore the potential association between antibiotic exposure and the development of depressive symptoms, we have designed a prospective cohort study in pregnant individuals. Adult pregnant individuals have been chosen for this study as they are a group who share some medical and biological characteristics, can be approached nationwide via targeted online media advertising and relevant health professionals (e.g., obstetricians, general medical practitioners, and midwives), and in whom approximately one in two individuals are exposed to antibiotics during their perinatal journey in Australia [8]. That is, like the general population, pregnant and postpartum individuals receive antibiotics for common infections of the skin and soft tissue and of the urinary, respiratory, and gastrointestinal tracts [9,10]. Additionally, a range of antibiotics (including penicillins, cephalosporins, aminoglycosides, macrolides, clindomycin, metronidazole, and vancomycin) is used in this population specifically to treat conditions such as chorioamnionitis [11] and preterm pre-labour rupture of membranes [12] and to prevent neonatal and/or maternal infections in individuals who are colonised with group B *Streptococcus* or who are undergoing caesarean section [13]. Furthermore, prior research exploring rates of depression during pregnancy and the postpartum period in Australia has reported rates of 5.9% to 8.9% during pregnancy (data from 2002 to 2016) [14,15] and between 8.1% and 9.5% up to 12 months postpartum (data from 2003 to 2005) [16].

### Aims

The overarching aim of this project is to investigate the effect of exposure to antibiotics during pregnancy and the postpartum period (i.e., up to 12 months after giving birth) on the development of depressive symptoms during pregnancy and up to 12 months postpartum.

## 2. Methods and Design

### 2.1. Study Design and Setting 

This study is an Australia-wide, prospective, longitudinal cohort study. Pregnant individuals will be recruited and complete four online surveys. Baseline data will be collected at the first survey when individuals are at least 32 weeks pregnant. Follow-up data are collected at surveys completed around 6 weeks, 6 months, and 12 months postpartum. A 12-month follow-up period was chosen considering the World Health Organization’s definition of postpartum depression as being depression occurring within 12 months post-delivery [17]. 

### 2.2. Inclusion and Exclusion Criteria

Pregnant individuals who reside in Australia and are 18+ years old will be eligible to participate. Participants will be excluded prospectively from this study if they are a surrogate or if upon birth their child/ren will be cared for by someone else either permanently or semi-permanently. They will be excluded retrospectively if they have a stillbirth or lose their child/ren for reasons such as miscarriage, prematurity, illness, or sudden infant death syndrome. 

### 2.3. Sample Size Calculation

The Peduzzi formula for multivariate analyses [18] was used to estimate the required sample size for this study. In Australia, the point prevalence of depressive symptoms has been previously reported to be 9.5% at 12 months postpartum (data from 2003 to 2005) [16]. Calculations were made conservatively assuming 10 potential predictor variables, including antibiotic use, in the final multiple logistic regression model; this would require a sample size of 1050 individuals. The recruitment target is 1500 to account for a drop-out rate of up to 30%. 

### 2.4. Recruitment

This study will be promoted both via online platforms (such as posts in pregnancy-related Facebook groups, e.g., Pregnancy Support Group Australia) and targeted paid advertising (via Facebook, Google, and Instagram), as well as via strategic flyer placement in family doctor, midwife, and obstetrician practices, pharmacies, radiology clinics, supermarkets, and pregnancy-associated service areas (e.g., hypnobirthing classes, studios specialising in yoga in pregnancy, etc.). Individuals can find out more about this study and enrol if interested by following the link on the advertisement/flyer or scanning a QR code. Participants will then be asked to read the electronic information sheet and provide consent on the electronic consent form. Prize draws will be used to encourage participation; there will be 41 chances to win a gift voucher for completing this study (with thirty AUD 50 vouchers, ten AUD 100 vouchers and one AUD 500 voucher on offer). Participants can opt to be included in the draw by clicking on a link at the end of their final survey; this link will record their preference separately from their survey responses.

## 3. Procedure

### 3.1. Data Collection

A browser-based metadata-driven capture system, Research Electronic Data Capture (REDCap), will be used for survey design and data collection. Data will be collected at four time points during an approximately 15-month period using online surveys. These time points will during the index pregnancy (at 32 or more weeks gestation) and at 6 weeks, 6 months, and 12 months postpartum. The first survey will be sent to participants when they are approximately 32 weeks pregnant or as soon as possible thereafter, according to the information they provide in their consent form. In this first survey, the participant’s estimated due date (EDD) will be captured. They will then receive an email two weeks before their EDD, asking if they have delivered. If not, another email will be sent to them 4 weeks later. Only two time points have been chosen for these prompts to avoid overburdening participants with correspondence. 

The remaining surveys will be sent to participants based on their recorded delivery date; this ensures participants can complete the following survey if they have missed prior ones. They will receive up to three reminders to start any survey. Piloting data indicate the surveys take approximately 25 to 35 min to complete. Considering this, participants will be able to save their responses midway and resume later using a unique allocated password. They will then be sent up to three reminder prompts to complete each survey. 

Data collected in the surveys will, where possible, be gathered using previously validated and/or used questions and scales (Table 1). The data collected will include demographic and socio-economic variables (age, education, employment, and income), medical and medication history (including history of personal and familial mental health disorders and antibiotic use), and data relating to factors previously identified as increasing the risk for developing depressive symptoms in general or specifically during and following pregnancy (Table 1 and Table 2). Individuals who report having delivered in a public hospital in Tasmania, Australia (where the study team is based) will be asked to provide additional consent for the team to access their medical records at the hospital they delivered at as well as from their local pharmacies. This additional step will be used to explore the accuracy of participants’ recall regarding antibiotic use. 

The development of depressive symptoms is defined as a new diagnosis of depression made and/or confirmed by a medical doctor and/or obtaining a score of 13 or more on the Edinburgh Postnatal Depression Scale (EPDS) [27]. For example, in the first survey (which is completed in the third trimester), participants are asked, “During your pregnancy, were you at any point diagnosed with depression? This question relates only to a diagnosis made or confirmed by a medical doctor (e.g., GP, psychiatrist, obstetrician)”, and in each follow-up survey (at 6 weeks, 6 months, and 12 months postpartum), individuals are asked, “Have you been newly diagnosed with depression since you answered the previous survey?” Additionally, participants will be asked to fill in the EPDS in each survey. A score of 13 or more is the standard cut-off score recommended for screening major depression and has been used by prior Australian and international studies [25,27,93,94,95,96,97,98]. Furthermore, the Royal Australian and New Zealand College of Obstetricians and Gynaecologists (RANZCOG) states that individuals with a score of 13 or more should be further reviewed, as this score may suggest a crisis [29]. 

In addition to the EPDS, all surveys will also include the Depression Anxiety Stress Scale (DASS-21) [31,32]. Unlike the EPDS, it assesses three aspects of mental health (depression, anxiety, and stress). Both validated tools are being used to allow collection of anxiety and stress symptoms, whilst also comparing the rate at which each tool identifies individuals requiring follow-up for depressive symptoms.

Information regarding antibiotic use will be collected by asking participants about their antibiotic intake. For example, in the first survey, the question is “Have you taken any courses of antibiotics during this pregnancy?” If they answer yes, then they will be asked for the name, number of courses, and the trimester in which they used the antibiotics. To reduce the potential for illnesses requiring antibiotics to bias the results, individuals will be asked prior to each survey whether they are using or have finished a course of antibiotics within the prior 14 days. If so, they will not be able to complete the survey until at least 14 days have passed following the self-reported expected date of completion for their antibiotics course. This will allow for recovery in the first week following antibiotics and then the assessment of their mental health state at the end of the second week (both the EPDS and DASS-21 ask individuals to respond by reflecting on their experiences in the prior 7 days). 

The consent form and the four surveys have been piloted and extensively reviewed by four pregnant and parous individuals, with varying levels of English fluency and education, prior to their implementation. Participant recruitment began at the end of August 2021. Data collection is ongoing and anticipated to be completed in early 2024.

### 3.2. Ethics Consideration

There is no significant risk posed to the individual or their infant(s) from participating in this observational study. In acknowledgement, however, that some of the factors explored in this study may evoke feelings of discomfort (e.g., questions relating to intimate partner abuse), individuals will be repeatedly reminded throughout the study documents and surveys that participation is voluntary, responses are strictly confidential, and none of the questions are compulsory. Furthermore, to encourage individuals to access support services if required, a statement will preface all questions exploring mental health and experience of intimate partner abuse, with contact details for relevant nationwide counselling and support services. These individuals will be provided contact information for nationally available round-the-clock services that provide both counsellor and peer support for individuals experiencing child loss.

Finally, the study platform will automatically identify and email individuals who either score 13 or more in the EPDS or whose responses to specific questions indicate they, or their child, may be at risk of harm, informing them that their responses suggest they may benefit from a discussion with their preferred healthcare provider regarding their current circumstances. This email will also provide contact details for nationwide organisations that provide support for individuals experiencing mental health crises, sexual assault, or family and domestic violence. 

### 3.3. Data Management

To protect participants’ privacy and identity, names and postal addresses will not be recorded for the main cohort; any data derived from consenting participants’ medication information from Tasmanian hospitals and pharmacies in the sub-cohort or address information for individuals who prefer to complete the survey using a hard copy will be securely stored in a location separate to their survey data. Furthermore, all EDD and delivery date-related data will be removed prior to data analysis and stored against participants’ study IDs in this same password-protected database located separately from the main database. All potentially identifying data will be permanently removed from all study databases following the completion of this study and the distribution of prize draws unless participants provide consent for future follow-up in the final survey. The email addresses of individuals who provide this additional consent will be recorded against their allocated study ID in a password-protected separate file stored in a separate location, accessible only to select members of the project team (M.P., M.T., M.W. (Mackenzie Williams), T.L., F.V., and C.M.).

### 3.4. Data Analysis and Plan

**Figure 1:**
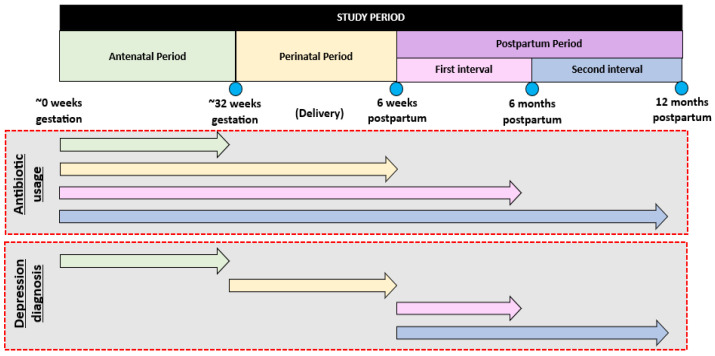
The period of antibiotic exposure in relation to the diagnosis of depression and measured depressive symptoms (via the EPDS). 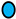 indicates the points of time when the Edinburgh Postpartum Depression Scale (EPDS) is used for screening depressive symptoms.

The primary objective is to explore whether antibiotic use is associated with the development of depressive symptoms. This will be explored during three time periods, as illustrated in Figure 1, and described below:

1. The antenatal period (from ~0 weeks gestation to mid-late third trimester);
2. The perinatal period (from mid-late third trimester to 6 weeks postpartum);
3. The postpartum period (from 6 weeks to 6 months postpartum and from 6 months to 12 months postpartum).

During the antenatal period, antibiotics used at any time during the index pregnancy will be explored as a potential predictor of depressive symptoms. The outcome variable will be depression diagnosed during the index pregnancy and/or an EPDS score of 13+ at the time of completing the first survey during the mid to late third trimester.

During the perinatal period, antibiotic use will be considered as any antibiotics that were received from ~0 weeks gestation to 6 weeks postpartum, including during caesarean section, labour, or hospitalisation. The outcome variable will be depression diagnosed after completion of the antenatal survey in the mid-late third trimester until 6 weeks postpartum and/or an EPDS score of 13+ at the time of completing the 6 weeks postpartum survey. Any individual diagnosed with depression during pregnancy and/or scored an EPDS score of 13+ in the antenatal survey will be marked as having had antenatal depressive symptoms in the index pregnancy.

During the postpartum period at two intervals, from 6 weeks to 6 months postpartum and from 6 months to 12 months postpartum, antibiotic use will be defined as having used antibiotics at any time during the index pregnancy up until the end of 6 months postpartum and 12 months postpartum, respectively. The outcome of interest will be defined as depressive symptoms (i.e., an EPDS score of 13+) at the time of completing the 6 months or 12 months postpartum survey and/or a new diagnosis of depression made or confirmed by a medical doctor in the period following the completion of the first postpartum survey (at approximately 6 weeks postpartum).

We acknowledge that collecting data at discrete time points rather than continuously creates some uncertainty regarding the relationship between the timing of antibiotic use and the timing of depressive symptoms. To address this issue, a sensitivity analysis will be conducted for each analysis using only an EPDS score of 13+ as the outcome measure, as the timing of the administration of the EPDS tool is clear in relation to antibiotic use that is disclosed in the same survey. In addition, the periodic nature of the surveys will allow us to exclude data from individuals whose depressive symptoms developed prior to any antibiotic use within the period of interest. For example, individuals who develop depressive symptoms during the antenatal period and then are treated with antibiotics for the first time within the study period in the peripartum period (e.g., for mastitis) will be excluded from the statistical analysis for the perinatal period and postpartum period, as their depressive symptoms preceded antibiotic use.

To explore the potential predictors of depressive symptoms, multiple logistic regression analyses will be used. The primary outcome of interest in these analyses will be the development of depressive symptoms, either as a new diagnosis made or confirmed by a medical doctor and/or an EPDS score of 13+. Table 2 contains a list of all variables that will be used in the univariate analyses. De-identified data will be analysed using SPSS (Statistical Package for Social Sciences, IBM^®^ Armonk, NY, USA). The Shapiro–Wilk Test of Normality and Normal Q-Q Plot will be used to test the data for normality numerically and graphically, respectively. If the data are approximately normally distributed, the following analyses will be conducted. Continuous variables will be summarised as means with standard deviations. The differences between groups will be tested using the chi-square test for categorical variables and independent *t*-test and one-way analysis of variance (ANOVA) for continuous variables (using Tukey’s HSD test post hoc if the data meet the assumption of homogeneity of variances, and Games Howell if not). Pearson’s rank coefficient will be calculated for measuring correlations between continuous variables. All variables with *p*-values ≤ 0.010 in the bivariate analyses will be included in the logistic regression analyses, after checking for collinearity and interdependence, where *p*-value ≤ 0.05 will indicate statistical significance. Findings will be summarised as adjusted odds ratios with 95% confidence intervals.

There are additional secondary objectives that will be explored in the analyses. Firstly, the incidence and pattern of postpartum depressive symptoms (diagnosis and symptoms) will be characterised. To account for participant withdrawal, person years of follow-up will also be reported for incidence of depressive symptoms. Secondly, there will be a comparison of the rates (and degree of overlap) with which individuals are identified using the EPDS and DASS-21 tools as experiencing symptoms of depression (defined as scoring 13+ in the EPDS, and 21+ in the depression component of the DASS-21).

## 4. Discussion 

### Strengths and Limitations of This Study

One of the main strengths of this study is the scope and comprehensiveness of the data collected. There has been one prior study exploring the effect of antibiotic exposure on the risk of developing depressive symptoms in this population; however, it was small (*n* = 120), very limited in the variables collected, and only considered antibiotics used during and within 14 days of delivery, despite reporting on their potential effect on mental health up to 6 months postpartum [76]. In contrast, our study is designed to be statistically powered to comprehensively investigate the potential association between antibiotic use during pregnancy, delivery, and up to 12 months postpartum and the development of depressive symptoms, taking into account a large range of relevant variables. 

There are, however, some limitations to this study. As discussed in the Data Analysis and Plan section, the collection of data at discrete time points introduces uncertainty when exploring a possible connection between antibiotic usage and depressive symptoms. To mitigate this limitation, a preliminary screening of the data will be conducted to exclude individuals who reported being diagnosed with depression and/or experiencing depressive symptoms prior to antibiotic use in previous surveys. Additionally, sensitivity analyses will be performed, focusing specifically on the EPDS as the outcome measure. This decision is based on the relatively clear timing of the administration of the EPDS tool in relation to antibiotic use, which is disclosed in the same survey. 

Other limitations include the potential for individuals who are experiencing symptoms of depression to be more likely to withdraw/drop out from this study, with the risk increasing with the severity of depression. Additionally, participants who are experiencing abuse or similarly significant risk factors for depression may choose to not answer relevant questions. Due to the online and confidential nature of this study, we will be unable to explore the reasons for these decisions or to identify if they are related to the development of depressive symptoms or other factors, such as time constraints. Another limitation is the reliance on individuals to provide accurate data and the possibility of under-reporting antibiotic use due to the busy nature of parenthood and/or the long period between surveys. To explore this potential limitation, we will compare recalled antibiotic use to antibiotic prescription and dispensing data in a sub-cohort of Tasmanian participants. Finally, while the survey collects data about recent (within 12 months) intimate partner abuse, it does not collect a comprehensive history regarding individuals’ experiences of abuse. This decision was made based on prior findings that recent experience of abuse is more strongly associated with symptoms of perinatal depression [99,100].

If this study identifies associations between antibiotic use and changes in mental health outcomes for participants, future clinical trials could be conducted to evaluate the impact of probiotics and/or prebiotics on mitigating these effects and on mental health in general. The results may also contribute to efforts already being made to reduce unnecessary antibiotic use worldwide.

## Figures and Tables

**Table 1:** Summary of proposed study variables at different time intervals.

Variable	Proposed Tool	Description of Tool	Pregnancy	Postpartum Period		
			Initial Survey	6-Week Survey	6-Month Survey	12-Month Survey
Demographics and Socioeconomic (incl. participant +/− partner work) status	MPUQ	The Maternal Health Study conducted by Murdoch Children’s Research Institute [19].	✔	✔	✔	✔
Current medical history and medication use *, including antibiotic and probiotic use	IDQ	N/A	✔	✔	✔	✔
Impact of COVID-19 pandemic on pregnancy experience	MPUQ	Modified questions from a study by Durankuş and Aksu [20].	✔	✔	✔	✔
Antenatal class attendance	IDQ	N/A	✔	✔	–	–
Physical activity	IDQ	N/A	✔	✔	✔	✔
Social media usage (non-work related)	IDQ	N/A	✔	✔	✔	✔
Pregnancy and fertility history	MPUQ	The Maternal Health Study conducted by Murdoch Children’s Research Institute [19].	✔	–	–	–
Pregnancy expectations and experience	MPUQ and IDQ	The Maternal Health Study conducted by Murdoch Children’s Research Institute [19].	✔	–	–	–
Delivery intention, expectation, and actual experience	MPUQ and IDQ	The Maternal Health Study conducted by Murdoch Children’s Research Institute [19].	✔	✔	–	–
Complications related to pregnancy/delivery	MPUQ	The Maternal Health Study conducted by Murdoch Children’s Research Institute [19].	✔	✔	–	–
Prior infant feeding experience	MPUQ and IDQ	The Maternal Health Study conducted by Murdoch Children’s Research Institute [19].	✔	–	–	–
Intended and actual infant feeding method(s)	MPUQ and IDQ	The Maternal Health Study conducted by Murdoch Children’s Research Institute [19].	✔	✔	✔	✔
Previous contraception use intended postpartum contraception use, and factors affecting use (e.g., personal +/− partner religion/cultural beliefs)	IDQ	N/A	✔	–	–	–
Actual postpartum contraception use	IDQ	N/A	–	✔	✔	✔
Pregnancy within 12 months of index pregnancy	IDQ	N/A	–	–	✔	✔
Knowledge about the postpartum period	IDQ	N/A	✔	–	–	✔
Stressful life events	MPUQ	Modified questions from The Social Readjustment Rating Scale [21].	–	–	–	✔
Prior and current substance use (incl. alcohol, smoking, and recreational drugs)	AUDIT-C, MPUQ and IDQ	AUDIT-C: Suggested by Australian Guide for the diagnosis of Fetal Alcohol Spectrum Disorder [22]. Provides a standardized method for the assessment of maternal alcohol use. Allows categorisation of the level of fatal risk associated with maternal drinking by derivation of the AUDIT-C score. The Maternal Health Study conducted by Murdoch Children’s Research Institute [19].	✔	–	–	✔ †
Mother-infant bonding	BPQ	Consists of a 25-item questionnaire [23]. Designed to detect disorders of the mother-infant relationship.	–	✔	✔	✔
Current intimate partner abuse	CAS-Short	Consists of 18 items for assessing emotional or physical abuse by a partner or ex-partner [24]. Explores history of abuse within the last 12 months. Developed and validated for use in general practices in Australia [24]. Used in previous studies conducted in Australia exploring factors affecting risk of developing depressive symptoms [16,25].	✔	–	–	✔
Sleep quality	SQS	Consists of a single item to measure overall sleep quality over the prior 7 nights [26].	✔	✔	✔	✔
Depression	EPDS	Consists of a 10-item questionnaire [27]. Widely used nationally and internationally [16,25,28]. Recommended by RANZCOG for screening for depression during perinatal period [29]. It has been used and validated for assessment of depression during pregnancy [30] and postpartum [27].	✔	✔	✔	✔
Depression, anxiety, and life stress	DASS-21	Consists of a 21-item questionnaire (7 questions for measuring each variable of depression, stress, and life stress) [31,32]. It is validated for use in the general population, but not specifically for assessing depression during pregnancy and postpartum [31,32].	✔	✔	✔	✔
Social support	OSSS-3	Consists of a 3-item questionnaire [33]. Measures level of social support [33].	✔	✔	✔	✔

IDQ = Investigator designed questionnaire, MPUQ = Modified previously used questionnaire, DASS-21 = Depression Anxiety Stress Scales; EPDS = Edinburgh Postnatal Depression Scale; OSSS-3 = Oslo Social Support Scale; SQS = Sleep Quality Scale; CAS-Short = The short version of the Composite Abuse Scale; PBQ = Postpartum Bonding Questionnaire; AUDIT-C = Alcohol Use Disorders Identification Test—Consumption; RANZCOG = the Royal Australian and New Zealand College of Obstetricians and Gynaecologists; N/A = Not applicable; * At the time of completing a survey, if a participant is taking a course of oral or intravenous antibiotics or has completed a course in the previous two weeks, the survey will be delayed for two weeks following their expected date of completion. This is to minimise potential bias introduced by having an illness requiring antibiotic prescription on EPDS and DASS-21 scores. Upon returning to the survey after the delay, they will be asked again about their use of antibiotics in the prior two weeks; their survey commencement will be delayed again if their prior use continues past the expected date of completion. ^†^ Only smoking status will be explored at 12 months postpartum.

**Table 2:** List of potential risk factors being investigated for depression during the pregnancy and postpartum periods.

Variables to Be Explored	Reference for Association with Depression in Prior Studies, Where Applicable	Variable Investigated during Mid-Late Third Trimester	Variable Investigated during Postpartum Time Points (6 Weeks, 6 Months, and 12 Months Postpartum)
Maternal age	[34,35,36]	✔	✔
Body Mass Index	[37,38,39]	✔	✔
Marital status	[40,41]	✔	
Socioeconomic status, maternal leave, partner maternal leave	[35,41,42,43,44,45,46,47,48]	✔	✔
Level of physical activity during pregnancy and postpartum	[49,50]	✔	✔
Smoking status, alcohol consumption, recreational drug use	[35,51,52]	✔	✔
Personal and family medical history (incl. depression, anxiety, other mental illness)	[36,41,43,46,53,54,55,56,57] and NVI	✔	✔
Nausea and vomiting during pregnancy	[58]	✔	N/A
Pre- and perinatal admission to hospital	[59]	✔	N/A
Whether the pregnancy was unplanned	[41,48,60,61,62,63,64,65]	✔	✔
Use of reproductive technologies	[57,66]	✔	✔
Gravidity and parity (incl. previous miscarriages/termination of pregnancy)	[48,60,64,65,67,68]	✔	✔
Whether they delivered in a private or public hospital	NVI	N/A	✔
Peri- and postpartum complications for mother and baby (e.g., painful caesarean section wound)	[54,59,62,69,70]	N/A	✔
Mode of delivery	[56,71,72,73]	N/A	✔
If the birth plan/preferences were able to be followed	NVI	N/A	✔
Length of stay in hospital postpartum	[74,75]	N/A	✔
Social support level	[43,46,48,60,63]	✔	✔
Medication use (e.g., antibiotic and probiotic, hormonal contraceptive use, antidepressant/anxiolytic use)	[76,77,78,79,80]	✔	✔
Number of other children to care for	[81]	✔	✔
Current partner abuse	[25,43,48,53,62,63,64,82]	✔	✔
Antenatal depression	[41,53]	-	✔
Symptoms of stress and anxiety	[43,55,56]	✔	✔
Stressful life events	[42,55,62]	-	✔
COVID-19 stress and psychological impact of pandemic	[20,83]	✔	✔
Sleep quality	[84,85]	✔	✔
Social media use	[86,87]	✔	✔
Feeding plans and outcome	[88,89]	✔	✔
Contraception	[80,90,91,92]	✔	✔

N/A = Not applicable; NVI = New variable for investigation.

## Data Availability

Data sharing is not applicable to this article.
